# From Valve Anatomy to Molecular Trajectories: Integrating Proteomics into Precision Care for Bicuspid Aortic Valve Disease

**DOI:** 10.3390/jcdd13070344

**Published:** 2026-07-22

**Authors:** Cheng Luo, Yugui Li, Wei Lu, Baoshi Zheng, Xiaoyong Xie

**Affiliations:** Department of Cardiovascular Surgery Ward, The First Affiliated Hospital of Guangxi Medical University, Nanning 530021, China; drluocheng@163.com (C.L.);

**Keywords:** bicuspid aortic valve, aortic stenosis, proteomics, aortopathy, mechanobiology, precision medicine

## Abstract

Bicuspid aortic valve (BAV) disease is a lifelong disorder in which congenital anatomy, tissue susceptibility, abnormal flow, and acquired fibrocalcific remodeling produce heterogeneous valve and aortic outcomes. This narrative review examined peer-reviewed literature indexed in PubMed/MEDLINE through June 2026 to evaluate how circulating proteomics could complement established imaging-based risk assessment. Published studies of incident aortic stenosis consistently implicate integrated stress, inflammation, apoptosis, and extracellular-matrix remodeling, with recurrent signals including GDF15, MMP12, and natriuretic peptides. These data support a long preclinical molecular phase, but existing proteomic models were developed predominantly in general aortic stenosis populations and cannot be transferred directly to BAV. We propose a five-layer framework integrating valve morphology and function, aortic phenotype and growth, flow and wall mechanics, molecular activity, and patient-specific lifetime context. In the near term, proteomics is best used for cohort enrichment, mechanistic phenotyping, and trial design rather than intervention decisions. Prospective BAV-specific cohorts, standardized imaging, repeated sampling, competing-risk analysis, external calibration, and demonstration of management-changing utility are required before clinical implementation. Molecular phenotyping should refine, not replace, guideline-based imaging and shared decision-making.

## 1. Introduction

Bicuspid aortic valve (BAV) is the most common congenital valvular anomaly and a major substrate for aortic valve intervention and thoracic aortic surgery in young and middle-aged adults. The deceptively simple definition—two functional leaflets rather than three—encompasses wide variation in cusp fusion, raphe morphology, commissural orientation, valve dysfunction, and aortic involvement [1]. Recent narrative reviews have comprehensively summarized BAV aortopathy, surgical decision-making, and CT-based assessment [2,3]. The less developed question is how dynamic molecular information might be integrated with anatomy and flow to identify an active disease trajectory before irreversible structural progression.

Guideline-based care appropriately relies on echocardiographic assessment of valve severity and ventricular response, cross-sectional imaging of the thoracic aorta, serial aortic measurements, family history, and established thresholds for intervention [4,5]. These measures are indispensable, but they identify established structural disease more readily than early biological activity. Consequently, patients with similar morphology and dimensions may still follow substantially different trajectories.

Aortic stenosis (AS) research has simultaneously moved toward high-dimensional molecular phenotyping. Population proteomics can measure thousands of circulating proteins and may reveal inflammatory, fibrotic, apoptotic, and hemodynamic signals before clinical diagnosis. This creates a translational question for BAV care: can molecular information be combined with anatomy and flow to detect high-risk trajectories earlier without overtesting patients whose disease will remain stable?

This review synthesizes BAV developmental biology, mechanobiology, multimodality imaging, and published proteomic evidence into a single framework. Its distinctive focus is the route from molecular association to BAV-specific validation and clinical utility. The objective is not to replace current thresholds, but to define how proteomics can be tested as an adjunct to lifelong, phenotype-guided management.

## 2. Literature Search Strategy

A targeted narrative search of PubMed/MEDLINE was performed through 22 June 2026. Search terms combined “bicuspid aortic valve”, “aortic stenosis”, “aortopathy”, “proteomics”, “biomarker”, “multi-omics”, “4D-flow MRI”, “wall shear stress”, “machine learning”, and “precision medicine”. English-language guidelines, systematic reviews, meta-analyses, prospective cohorts, imaging studies, tissue studies, and mechanistically informative experimental reports were prioritized. Reference lists of key articles were also screened. Because the purpose was conceptual synthesis rather than estimation of a pooled effect, no formal risk-of-bias instrument or meta-analysis was applied. Evidence derived from general AS populations was explicitly separated from evidence obtained in patients with BAV.

A narrative rather than a systematic or scoping review format was deliberately chosen. The objective was conceptual integration across developmental biology, mechanobiology, multimodality imaging, and population proteomics—domains that do not share a single, quantifiable research question amenable to systematic synthesis or meta-analytic pooling. Consistent with recommended narrative-review practice, articles were included if they were peer-reviewed, English-language reports of original data, guidelines, or reviews addressing BAV or AS anatomy, hemodynamics, biomarkers, or management; conference abstracts, non-English-language reports, and case reports were excluded. Because the objective was conceptual synthesis rather than quantitative estimation, a formal PRISMA-style record of the numbers of records identified, screened, and excluded at each stage was not kept; this trade-off is acknowledged as a limitation in Section 10.

## 3. BAV as a Lifelong Systems Disease

### 3.1. Developmental and Genetic Substrate

BAV is best understood as a disorder of cardiovascular development rather than an isolated leaflet defect. Semilunar valve formation depends on endocardial cushion remodeling, endothelial-to-mesenchymal transition, neural crest and second heart field contributions, extracellular-matrix turnover, and coordinated commissural formation. Perturbation of these programs can produce cusp fusion and may simultaneously influence the proximal aortic media. Familial clustering and enrichment among first-degree relatives support inherited susceptibility, although nonsyndromic BAV is usually polygenic, and routine genetic testing has limited sensitivity [6,7].

NOTCH1 remains the most extensively studied gene linking abnormal valve development with later calcification. SMAD6, ROBO4, GATA-family factors, and transforming growth factor-beta pathways have also been implicated, but no single variant explains most clinical disease [8,9,10]. The practical consequence is that genotype currently supplements rather than replaces family screening and phenotypic surveillance. Genetic architecture nevertheless establishes a tissue substrate on which decades of mechanical and metabolic exposure act.

### 3.2. Valve Degeneration and Aortic Wall Vulnerability

Calcific BAV stenosis is an active fibrocalcific process. Endothelial injury permits lipid infiltration and inflammatory recruitment; valvular interstitial cells adopt myofibroblastic and osteogenic phenotypes; extracellular matrix is reorganized; and progressive mineralization stiffens the leaflets. BAV geometry amplifies this biology by creating nonuniform strain and turbulent transvalvular flow. Thus, congenital anatomy and acquired degeneration are sequentially linked rather than competing explanations.

BAV-associated aortopathy is similarly multifactorial. Elastic fiber fragmentation, smooth muscle cell dysfunction, collagen remodeling, and matrix metalloproteinase activity may reduce wall resilience. Eccentric jets and regionally abnormal wall shear stress then act on a susceptible aorta, producing phenotype-specific remodeling. Human 4D-flow magnetic resonance studies associate cusp-fusion pattern, flow displacement, regional wall shear stress, and aortic wall changes [11,12,13]. Intrinsic vulnerability and altered hemodynamics should therefore be viewed as interacting components of one disease system (Table 1).

## 4. Why Dynamic Molecular Phenotyping Is Attractive

Anatomy is relatively stable; biological activity is not. A circulating protein can reflect active inflammation, endothelial stress, matrix turnover, ventricular load, renal handling, or systemic aging at the time of sampling. This dynamic quality is both the promise and the challenge of proteomics. A well-validated panel might identify an active trajectory before conventional imaging changes, but a poorly specified panel may merely reproduce age, comorbidity, renal function, or generalized cardiovascular risk.

The conceptual advantage is particularly relevant to BAV. Two patients with similar cusp fusion and aortic diameter may have different tissue susceptibility, flow exposure, and rates of progression. Molecular measures could help distinguish a stable phenotype from one in which endothelial injury, apoptosis, or matrix remodeling is already active. Importantly, this information should be interpreted alongside imaging; a biomarker alone cannot determine whether the dominant threat is valvular stenosis, regurgitation, aortic enlargement, or myocardial decompensation.

Genetic scores estimate inherited propensity and remain essentially fixed across life. Proteomic profiles integrate inherited susceptibility with age, environment, hemodynamic load, and current disease activity. In AS, polygenic scores add modest discrimination to clinical factors, whereas large-scale proteomic studies have identified reproducible risk signals and multi-protein patterns [14,15,16,17]. Whether this advantage persists after BAV-specific imaging variables are added remains unknown.

## 5. Proteomic Signals Preceding Aortic Stenosis

### 5.1. Published Population-Scale Evidence

Published prospective studies have demonstrated that circulating proteins can predict incident AS years before valve replacement or clinical recognition. Recurrent signals include growth differentiation factor 15 (GDF15), macrophage metalloelastase (MMP12), and natriuretic peptides, while multi-protein panels add information beyond conventional risk factors in selected cohorts [15,16,17]. Targeted serum proteomics and valve-tissue proteomic/degradomic studies further implicate immune activation, extracellular-matrix turnover, fibrosis, and calcification [18,19].

These findings support a prolonged molecular phase in which systemic stress, macrophage activation, matrix injury, and myocardial load evolve before advanced obstruction. However, individual proteins vary in valve specificity. GDF15 and NT-proBNP may largely reflect integrated cardiovascular stress rather than valve-specific pathology. In particular, GDF15 is not specific even to cardiovascular disease: it is a stress-responsive cytokine that also rises with aging, chronic kidney disease, cancer, and other systemic inflammatory states, so its elevation in AS cohorts most likely reflects generalized biological stress rather than a valve-specific signal. MMP12 and tissue-derived extracellular-matrix signatures, by contrast, are mechanistically closer to leaflet remodeling. The active-disease model is consistent with the established concept of AS as a disorder of both the valve and the myocardium rather than passive age-related wear [20].

The central translational opportunity is therefore not a universal “AS blood test”, but a biologically interpretable panel that adds information to imaging at a defined disease stage. Any candidate panel must be evaluated for calibration, repeatability, specificity for valve versus systemic disease, and incremental value over simpler biomarkers.

### 5.2. Mechanistic Organization of Published Signals

Viewed mechanistically, the published signals span cellular stress, immune activation, myocardial load, and extracellular-matrix remodeling, with differing relevance to valve and aortic pathology in BAV (Table 2).

### 5.3. From Association to Mechanism

Circulating associations become clinically meaningful only when linked to local tissue biology. Transcriptomic studies of calcified bicuspid, tricuspid, and normal valves demonstrate phenotype-dependent inflammatory, extracellular-matrix, and osteogenic programs [22]. More recent multi-omics work has identified candidate regulatory nodes in BAV calcification, including TRPV2, but these observations remain mechanistic and require prospective clinical validation [23].

The next step is triangulation across plasma, valve tissue, imaging, and perturbation experiments. Candidate proteins should be localized to specific valve cell populations, tested under BAV-relevant shear and strain, and evaluated longitudinally against prespecified structural outcomes. Concordance across these levels would strengthen causal interpretation and help distinguish mediators from epiphenomena.

## 6. Translating Proteomics to BAV: Opportunity Without Overreach

### 6.1. Why General AS Models Cannot Simply Be Imported

This limitation is conceptual as well as statistical: BAV should not be regarded merely as aortic stenosis occurring on a two-cusp valve, but as a biologically distinct entity in which developmental origin, tissue susceptibility, and disturbed flow diverge from tricuspid AS from the outset (Section 3.1 and Section 3.2). Consequently, proteomic associations established in predominantly tricuspid AS cohorts cannot be assumed to generalize to BAV without dedicated validation. Existing proteomic models were derived mainly in general AS populations and often used electronic health record outcomes. They did not systematically establish BAV morphology, raphe calcification, commissural geometry, 4D-flow metrics, or serial aortic growth. Moreover, recent BAV studies combining inflammatory or metabolic markers with cohort data and explainable machine learning suggest that BAV-specific predictors may differ from general AS signatures [24,25]. Direct transfer of a general AS model would therefore be premature.

BAV also creates competing trajectories. A patient may require surgery for aortic regurgitation or aortopathy before stenosis develops; these events can censor or alter apparent AS risk. Surgical referral, imaging intensity, and age distributions differ from community cohorts. A BAV-specific model must use adjudicated outcomes, account for competing interventions, and test whether proteins add information beyond high-quality imaging and family history.

### 6.2. Near-Term Research Uses

Before clinical adoption, proteomics can strengthen BAV research in three ways. First, it can enrich longitudinal cohorts for individuals likely to show measurable progression, improving the efficiency of advanced imaging and mechanistic studies. Second, it can identify biological subtypes, for example, a stress-dominant profile involving GDF15 and natriuretic peptides, an immune pattern detected by targeted serum proteomics, or a matrix-remodeling profile involving MMP12 and tissue-derived extracellular-matrix signatures. Third, paired plasma, imaging, and surgical tissue studies can connect circulating signals to regional flow and tissue pathology.

**Figure 1:**
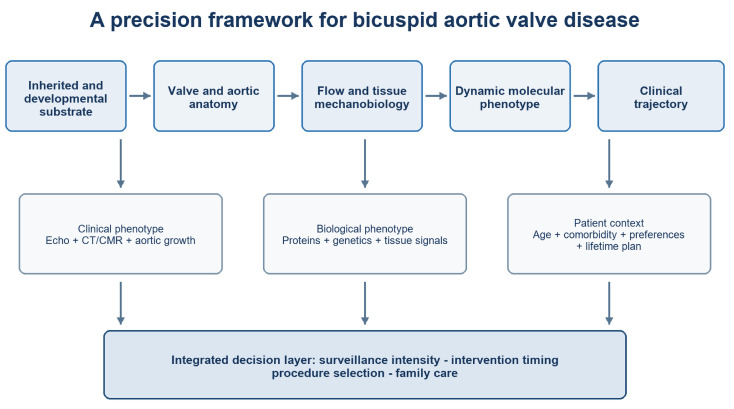
Proposed integration of structural, hemodynamic, molecular, and lifetime data in BAV. Molecular phenotyping is an adjunctive layer for identifying biological activity and refining research stratification; it does not replace guideline-based imaging or established intervention thresholds.

The most informative design would not ask whether a protein predicts a diagnosis code. It would ask whether repeated molecular profiles predict prespecified BAV outcomes: progression of peak velocity or valve calcification; new or worsening AR; indexed aortic growth; regional wall-shear abnormalities; myocardial fibrosis; or need for valve and/or aortic intervention. Such intermediate phenotypes may be biologically closer to the measured proteins and more actionable than a single composite endpoint (Figure 1).

The organizing contribution of this framework is not the five-layer taxonomy itself, which recapitulates domains already summarized in Table 1 (morphology, function, ventricular response, aortic phenotype, flow, molecular phenotype), but the staged decision logic it enables: molecular activity is proposed as a trigger for confirmatory imaging escalation, not as an independent, parallel risk layer (see Section 7.2). This staging logic, rather than the layer structure itself, is intended as the paper’s principal conceptual contribution.

## 7. An Integrated Precision Pathway

### 7.1. Baseline Phenotyping

Every patient should first receive a complete conventional phenotype: cusp fusion and raphe, valve function, ventricular size and function, root and ascending aortic dimensions, blood pressure, coarctation and associated congenital lesions, family history, and symptoms. Transthoracic echocardiography remains the cornerstone. CT or cardiovascular magnetic resonance is appropriate when the aorta is incompletely visualized, measurements are discordant, anatomy is complex, or intervention is planned [26,27].

A complete baseline also establishes the outcome context for future biomarkers. A high protein score in a patient with severe established stenosis may reflect downstream hemodynamic stress, whereas the same score in a young patient with mild dysfunction could represent early biological activity. Biomarker interpretation without stage and phenotype is therefore unsafe.

### 7.2. Longitudinal Surveillance

Surveillance intensity should continue to be driven by valve severity, ventricular response, aortic size and growth, symptoms, and recognized risk modifiers. Advanced flow imaging may be considered in research or selected discordant cases, especially when regional aortic remodeling is unexplained by diameter alone. Serial measurements should be performed with consistent modality, plane, and convention whenever possible.

In a future validated model, repeated proteomics could be used as a trigger for confirmatory imaging rather than as a stand-alone decision rule. A rising matrix/inflammatory score might prompt earlier CT calcium assessment or CMR; a low, stable score might support standard rather than intensified follow-up. Such strategies must be tested prospectively because apparent rule-out performance in a low-incidence population can be misleading if calibration changes across age, sex, ancestry, renal function, or assay platform.

### 7.3. Intervention and Lifetime Planning

Intervention remains anchored in symptoms, severity of AS or aortic regurgitation, ventricular consequences, aortic dimensions and growth, and procedural anatomy. Surgical aortic valve replacement remains central for many patients, particularly younger individuals and those requiring concomitant aortic repair. Valve repair, valve-sparing root replacement, or the Ross procedure may be appropriate in selected patients at experienced centers. Transcatheter aortic valve replacement is feasible in selected BAV anatomy, especially in older or higher-risk patients, but raphe calcification, asymmetric expansion, coronary access, paravalvular regurgitation, durability, and progressive aortopathy require careful lifetime planning [28,29,30,31].

No proteomic profile currently justifies earlier intervention outside established criteria. Its plausible future role is to identify a high-activity state for closer surveillance or trial enrollment. A biomarker would need to show that acting on it improves patient-important outcomes, not merely that it predicts them (Table 3).

## 8. Validation Agenda for BAV-Specific Proteomics

Several factors explain why circulating proteomic biomarkers have not yet been established in routine BAV or AS practice. Published signals derive from heterogeneous analytical platforms—affinity-based panels (e.g., Olink, SomaScan) and mass-spectrometry-based discovery differ in epitope specificity, dynamic range, and susceptibility to matrix effects, and serum versus plasma sampling can materially change measured concentrations—so methodological differences across studies limit direct comparability and may explain discordant effect sizes for the same protein. In addition, almost no signal has been replicated in an independently ascertained, BAV-specific cohort with adjudicated imaging outcomes, and no trial has yet tested whether acting on a proteomic result, rather than merely predicting an outcome, improves patient-important endpoints; without such utility data, regulatory and reimbursement pathways for clinical adoption cannot be established. Assay costs, turnaround times, and the absence of harmonized reporting standards across platforms further slow translation into guideline-based care.

A clinically credible BAV biomarker program requires staged validation. Analytical validity comes first: assay precision, batch stability, missingness, and comparability of plasma and serum must be established using accepted cardiovascular proteomics principles [32]. Clinical validity then requires external cohorts with adjudicated BAV morphology and outcomes. Models should be evaluated with discrimination, calibration, reclassification, decision-curve analysis, and competing-risk methods rather than area under the curve alone.

The reference clinical model must be strong. Age and comorbidity are insufficient comparators for BAV; the benchmark should include valve morphology and function, ventricular measures, aortic dimensions and growth, calcification, family history, and relevant procedural anatomy. Automated hemodynamic analysis, phenotype-specific 4D-flow assessment, and patient-informed simulations illustrate the rapidly improving imaging comparator against which molecular markers must add value [33,34,35]. Repeated protein measurements may be more informative than a single baseline value, but increase cost and complexity.

Finally, clinical utility requires a management strategy. A trial could randomize patients with mild-to-moderate BAV disease to standard surveillance or biomarker-informed imaging intervals, with prespecified safety boundaries. Alternative designs could use the panel to enrich prevention trials targeting lipoprotein(a), inflammation, fibrosis, or matrix remodeling. Any such program must monitor false reassurance, incidental risk labeling, anxiety, access inequity, and ancestry-specific calibration (Table 4).

## 9. Therapeutic Implications

Published proteomic evidence reinforces several candidate therapeutic axes. GDF15 and natriuretic peptides indicate integrated stress but are unlikely to be valve-specific targets. MMP12 and extracellular-matrix signatures point toward macrophage-mediated matrix degradation and fibrosis. Tissue and multi-omics studies additionally implicate senescence, metabolism, and osteogenic signaling. These pathways should be treated as target hypotheses rather than therapeutic claims.

Biological plausibility does not guarantee therapeutic efficacy. Trials targeting advanced calcification have generally been disappointing, possibly because intervention occurs after self-perpetuating mineralization and fibrosis are established [36]. A validated early molecular phenotype could identify the window in which a pathway-directed therapy is most likely to work. This is the key attraction of mechanism-linked risk prediction: the same panel may enrich both the appropriate patient and disease stage, including patients with moderate disease who carry substantial long-term burden [37].

BAV-specific therapy will probably require more than one axis. The dominant process may differ between a young patient with AR and root dilation, a middle-aged patient with progressive calcific stenosis, and an older patient with mixed valve disease and ventricular fibrosis. Molecular subtyping should therefore be outcome-specific and combined with anatomical phenotype rather than treated as a universal BAV score.

## 10. Limitations of the Current Evidence

The principal limitation is transferability. Most proteomic evidence concerns incident AS in general populations, not longitudinally phenotyped BAV. Electronic health record codes favor specificity for clinically recognized disease but miss mild or asymptomatic stenosis, a persistent challenge in community valve-disease detection [38]. Healthy-volunteer bias and predominantly European ancestry can distort absolute risk. Publication bias is an additional concern: studies reporting positive or novel proteomic associations are more likely to be published than null results, which may inflate the apparent strength and consistency of candidate signals such as GDF15 and MMP12. Proteomic signals are influenced by age, renal function, systemic inflammation, cardiovascular comorbidity, and preanalytical factors. Tissue validation from surgical valves represents advanced disease and cannot, by itself, establish early causality.

BAV has a long and heterogeneous natural history, with valve dysfunction, aortic enlargement, and intervention acting as competing outcomes [39,40,41,42]. Current molecular findings should therefore be considered hypothesis-generating for BAV. Clinical decisions require calibrated absolute risk in the intended population, external replication, and evidence that acting on a result improves patient-important outcomes. This narrative review also did not use systematic review methods and may be affected by selection bias despite the targeted search strategy.

## 11. Conclusions

BAV is a lifelong systemic disease in which developmental susceptibility, abnormal valve and aortic anatomy, disturbed flow, tissue vulnerability, and acquired fibrocalcific remodeling interact. Current imaging-based care remains the foundation of safe management, but it does not fully capture biological activity before structural progression becomes apparent.

Large-scale proteomics shows that AS is preceded by a molecular trajectory involving cellular stress, inflammation, and matrix remodeling. Published signals provide a plausible translational bridge to BAV, but the correct next step is prospective BAV-specific validation integrated with high-quality imaging, competing-risk analysis, mechanistic experiments, and trials of biomarker-guided surveillance or therapeutic enrichment. Precision BAV care should become multilayered while remaining clinically grounded: molecular signals should refine the interpretation of anatomy, not replace it.

## Figures and Tables

**Table 1:** Established precision domains in BAV and the residual biological gap.

Domain	Representative Measures	Current Value	Unresolved Gap
Valve morphology	Fusion pattern, raphe, commissural angle, calcium distribution	Diagnosis; repair/TAVR feasibility	Does not directly measure biological activity
Valve function	AS/AR severity, gradients, valve area, regurgitant mechanism	Follow-up and intervention timing	May become abnormal after prolonged subclinical remodeling
Ventricular response	Volumes, ejection fraction, strain, fibrosis	Detects downstream myocardial consequences	May reflect late-stage consequences rather than initiating disease
Aortic phenotype	Root/ascending/arch dimensions and growth	Surveillance and surgical planning	Diameter incompletely captures wall vulnerability
Flow phenotype	Jet eccentricity, helicity, vortex, wall shear stress	Explains regional loading; may refine risk	Acquisition and thresholds are not standardized
Molecular phenotype	Genetic susceptibility and circulating/tissue proteins	Potential early activity and mechanistic subtype	BAV-specific prospective validation is lacking

Abbreviations: AS, aortic stenosis; AR, aortic regurgitation; TAVR, transcatheter aortic valve replacement.

**Table 2:** Published proteomic signals in aortic stenosis and their relevance to BAV research.

Protein or Signature	Published Evidence	Biological Domain	Interpretation for BAV
GDF15	Prospective incident-AS cohorts [15,16]	Integrated cellular stress and inflammation	Strong risk signal, but limited valve specificity
MMP12	Incident-AS and calcification studies [15]	Macrophage activity and matrix degradation	Plausible bridge between inflammation and leaflet/aortic remodeling
NT-proBNP/NPPB	Clinical and proteomic cohorts [15,16,17,21]	Myocardial wall stress	Predominantly downstream; useful for context rather than valve specificity
C1QTNF1	Large-scale association and causal-prioritization analyses [15]	Inflammatory and metabolic signaling	Requires replication and phenotype-specific validation in BAV
Immune-protein pattern	Targeted serum proteomics [18]	Immune activation	May identify an inflammatory subtype; susceptible to systemic confounding
ECM proteome/degradome	Calcified-valve tissue studies [19]	Collagen, elastin, and matrix turnover	Potential source of tissue-informed circulating markers

Abbreviations: AS, aortic stenosis; BAV, bicuspid aortic valve; GDF15, growth differentiation factor 15; MMP12, matrix metalloproteinase-12 (macrophage metalloelastase); NT-proBNP/NPPB, N-terminal pro-B-type natriuretic peptide/natriuretic peptide precursor B gene; C1QTNF1, C1q/TNF-related protein 1; ECM, extracellular matrix.

**Table 3:** Proposed clinical-research pathway for molecular precision in BAV.

Step	Inputs	Purpose	Current Status
1. Confirm phenotype	Echo; CT/CMR when needed; family history; BP; symptoms	Establish valve, ventricle, aorta, and lifetime context	Standard care
2. Define activity	Serial imaging; optional research proteomics and 4D-flow	Separate stable anatomy from active remodeling	Research adjunct
3. Classify trajectory	Valve progression, aortic growth, myocardial response, competing events	Choose outcome-specific surveillance	Prospective validation
4. Escalate evaluation	A validated high-risk molecular signal; earlier confirmatory imaging	Detect actionable structural change	Requires a utility trial
5. Decide on the intervention	Guideline criteria plus anatomy, age, preference, and center expertise	Select surgery, repair, valve-sparing, Ross, or TAVR	Standard care; biomarker not determinative
6. Continue lifelong care	Post-procedure aortic/valve surveillance and family screening	Address residual and familial risk	Standard care

Abbreviations: BAV, bicuspid aortic valve; CT, computed tomography; CMR, cardiovascular magnetic resonance; BP, blood pressure; TAVR, transcatheter aortic valve replacement.

**Table 4:** Minimum evidence needed before clinical implementation.

Evidence Domain	Required Work	Decision Answered
Analytical	Reproducibility, assay drift, specimen type, platform transfer	Can the panel be measured reliably?
Population	Prospective BAV cohorts across age, sex, ancestry, and geography	Does performance generalize?
Phenotype	Core-lab imaging and adjudicated valve/aortic outcomes	What exactly does the panel predict?
Incremental value	Comparison with full imaging-based clinical models	Does proteomics add useful information?
Calibration	Absolute-risk calibration and competing-risk assessment	Are predicted risks trustworthy?
Mechanism	Valve-cell, shear-stress, tissue, and causal experiments	Are markers mediators or correlates?
Utility	Biomarker-guided surveillance or trial-enrichment studies	Does acting on the result improve outcomes?
Implementation	Cost, turnaround, interpretability, equity, privacy	Can the strategy be deployed responsibly?

Abbreviations: BAV, bicuspid aortic valve.

## Data Availability

No new data were created or analyzed in this review. Data sharing does not apply to this article.
